# Dysregulated Notch Signaling in the Airway Epithelium of Children with Wheeze

**DOI:** 10.3390/jpm11121323

**Published:** 2021-12-07

**Authors:** Thomas Iosifidis, Erika N. Sutanto, Samuel T. Montgomery, Patricia Agudelo-Romero, Kevin Looi, Kak-Ming Ling, Nicole C. Shaw, Luke W. Garratt, Jessica Hillas, Kelly M. Martinovich, Elizabeth Kicic-Starcevich, Shyan Vijayasekaran, Francis J. Lannigan, Paul J. Rigby, Darryl A. Knight, Stephen M. Stick, Anthony Kicic

**Affiliations:** 1 Wal-yan Respiratory Research Centre, Telethon Kids Institute, Perth, WA 6009, Australia; thomas.iosifidis@telethonkids.org.au (T.I.); Erika.Sutanto@telethonkids.org.au (E.N.S.); samuel.montgomery@telethonkids.org.au (S.T.M.); Patricia.AgudeloRomero@telethonkids.org.au (P.A.-R.); Kevin.Looi@telethonkids.org.au (K.L.); kak-ming.ling@telethonkids.org.au (K.-M.L.); nicole.shaw@telethonkids.org.au (N.C.S.); luke.garratt@telethonkids.org.au (L.W.G.); jessica.hillas@telethonkids.org.au (J.H.); Kelly.Martinovich@telethonkids.org.au (K.M.M.); liz.starcevich@telethonkids.org.au (E.K.-S.); stephen.stick@health.wa.gov.au (S.M.S.); 2 Centre for Cell Therapy and Regenerative Medicine, School of Medicine, The University of Western Australia, Nedlands, WA 6009, Australia; 3 School of Population Health, Curtin University, Bentley, WA 6102, Australia; 4 School of Molecular Science, The University of Western Australia, Nedlands, WA 6009, Australia; 5 Division of Paediatrics, Medical School, The University of Western Australia, Nedlands, WA 6009, Australia; shyan.vijayasekaran@health.wa.gov.au (S.V.); Francis@nedlandsent.com.au (F.J.L.); 6 School of Medicine, Notre Dame University, Fremantle, WA 6160, Australia; 7 Centre of Microscopy, Characterisation and Analysis, The University of Western Australia, Nedlands, WA 6009, Australia; paul.rigby@uwa.edu.au; 8 School of Biomedical Sciences and Pharmacy, University of Newcastle, Newcastle, NSW 2308, Australia; dknight2@providencehealth.bc.ca; 9 Priority Research Centre for Healthy Lungs, Hunter Medical Research Institute, Newcastle, NSW 2305, Australia; 10 Department of Anesthesiology, Pharmacology and Therapeutics, University of British Columbia, Vancouver, BC V6H 3Z6, Canada; 11 Department of Respiratory and Sleep Medicine, Perth Children’s Hospital, Nedlands, WA 6009, Australia

**Keywords:** pediatrics, wheeze, airway epithelium, wound repair, Notch

## Abstract

The airway epithelium of children with wheeze is characterized by defective repair that contributes to disease pathobiology. Dysregulation of developmental processes controlled by Notch has been identified in chronic asthma. However, its role in airway epithelial cells of young children with wheeze, particularly during repair, is yet to be determined. We hypothesized that Notch is dysregulated in primary airway epithelial cells (pAEC) of children with wheeze contributing to defective repair. This study investigated transcriptional and protein expression and function of Notch in pAEC isolated from children with and without wheeze. Primary AEC of children with and without wheeze were found to express all known Notch receptors and ligands, although pAEC from children with wheeze expressed significantly lower *NOTCH2* (10-fold, *p* = 0.004) and higher *JAG1* (3.5-fold, *p* = 0.002) mRNA levels. These dysregulations were maintained in vitro and cultures from children with wheeze displayed altered kinetics of both *NOTCH2* and *JAG1* expression during repair. Following Notch signaling inhibition, pAEC from children without wheeze failed to repair (wound closure rate of 76.9 ± 3.2%). Overexpression of *NOTCH2* in pAEC from children with wheeze failed to rescue epithelial repair following wounding. This study illustrates the involvement of the Notch pathway in airway epithelial wound repair in health and disease, where its dysregulation may contribute to asthma development.

## 1. Introduction

In children, wheezing illnesses and asthma exacerbations are some of the most common causes of hospital presentation with current treatments targeting symptoms (e.g., bronchoconstriction and airway inflammation) and not the underlying mechanisms. Despite these treatments, young children experience recurrent and severe wheezing that may develop in chronic asthma in later childhood and adulthood. Thus, there is a medical need for targeted treatments to modify disease trajectories in early life. The airway epithelium has been highlighted as an important contributor to asthma development through the identification of epithelial-specific genes that associate with early-onset asthma in various GWAS studies [1,2,3]. Furthermore, functional assessment of airway epithelial cells in vitro by our team and others have observed intrinsic vulnerabilities that include dysregulated reparative capacity and loss of barrier integrity with recurrent pre-school wheeze and school-aged asthma [4,5,6,7,8,9,10,11]. Impaired airway epithelial function following insults has been associated with respiratory exacerbations [11,12] and drives chronic airway inflammation and remodelling [13,14,15]. Thus, understanding the underlying disease mechanisms that dysregulate airway epithelial repair in early childhood could identify new areas for targeted therapies to halt wheeze recurrence and prevent asthma development. 

Several studies have indicated important parallels between the epithelial reparative processes controlled by Notch and extracellular matrix–integrin interactions. For example, Notch signaling has been found to directly control α5β1 integrin activation and subsequent adhesion of mammalian epithelial cells to fibronectin [16]. Furthermore, pharmacological or genetic modulation of Notch signaling impacts on epithelial cell migration and repair capacity [17,18]. Previous investigations have demonstrated dysregulation of Notch [19,20], fibronectin [21] and integrin [11] pathways in airway epithelial cells from children and adults with wheeze or asthma to negatively affect airway epithelial homeostasis and repair response to injury. 

In vertebrates, there are four different Notch receptors, referred to as Notch(1–4) and a number of Notch-binding ligands called Delta-like (DLL1, 3 & 4) and Jagged (JAG1–2) [22]. Interactions between the Notch receptors and their ligands occur at 1:1 stoichiometric ratio and are critical in modulating gene transcription and cellular functions, including cell proliferation, migration and differentiation [23,24,25]. However, there remains a paucity of data on the role of Notch signaling during wound repair, particularly in the context of the airway epithelium from young children in health and disease. 

In this study, we investigated the gene expression profile of Notch receptors and ligands in the airways of children with and without wheeze and tested the hypothesis that dysregulated Notch pathways adversely affect airway epithelial cell reparative capacity. We profiled gene expression of Notch receptors and ligands in ex vivo lower airway epithelial cell samples from children with and without wheeze. In addition, we examined the expression kinetics of Notch during wound repair and assessed the effect of Notch inhibition on pAEC wound repair in vitro. Finally, Notch2 overexpression was achieved in pAEC from children with wheeze where proliferation and wound repair capacity of pAEC cultures were assessed against retroviral vector and wildtype controls.

## 2. Materials and Methods

### 2.1. Reagents

Bovine serum albumin, fetal bovine serum, bovine hypothalamus acetone power, hydrocortisone, recombinant human epidermal growth factor, epinephrine hydrochloride, fibronectin, rat-tail type I collagen, triiodothyronine, transferrin, trans-retinoic acid, trypsin, gentamicin and N-(N-[3,5-difluorophenacetyl]-l-alanyl)-S-phenylglycine t-butyl ester (DAPT) were obtained from Sigma-Aldrich (St. Louis, MO, USA), fibronectin (BD, Franklin Lakes, NJ, USA), Y-27632 ROCK inhibitor (Enzo Life Sciences, Farmingdale, NY, USA) and all tissue culture plastic ware were purchased from Corning (Corning, NY, USA). Bronchial epithelial basal medium (BEBM™) and bronchial epithelial cell growth medium (BEGM™) were purchased from LONZA (Melbourne, VIC, Australia). Penicillin G, RPMI-1640 media, streptomycin sulfate, amphotericin B and L-glutamine were purchased from Invitrogen (Carlsbad, CA, USA). Ultroser G was supplied from Ciphergen Technologies (Fremont, CA, USA). For retroviral plasmid construct, propagation and replication-deficient retrovirus generation, DMEM and F12 culture media were purchased from Gibco (Melbourne, VIC, Australia). Restriction enzymes (BamHI, HindIII, NotI and PspOMI), T4 DNA ligase and NEB^®^ 10-beta competent *E. coli* cells were purchased from New England Biolabs (Ipswich, MA, USA). The transfection reagent FuGene 6 was purchased from Promega (Madison, WI, USA) Corporation (Madison, WI, USA). MSCV-IRES-GFP was kindly provided by Martine Roussel and Arthur Nienhuis of St Jude Children’s Research Hospital (Memphis, TN, USA) and pcDNA3.1 Notch2.GFP plasmid was purchased from GenScript (Piscataway, NJ, USA). RT-PCR and qPCR reagents were sourced from Thermo Fisher Scientific. Antibodies against human Notch2 extracellular (monoclonal rabbit, clone D67C8, #4530; Cell Signaling Technologies, Danvers, MA, USA) and intracellular (monoclonal mouse, clone 602845, MAB37351; R&D Systems) domains, Jagged1 (polyclonal goat, AF1277; R&D Systems), were also obtained. 

### 2.2. Study Participants and Cell Types

Two cohorts were used in this study: 20 (17 male) children with persistent wheeze, and 26 (13 male) children with no history of wheeze (Table S4). The study involved subjects between 1.2 and 15.6 years of age undergoing elective surgery for non-respiratory related conditions. Furthermore, none had clinical symptoms of bacterial or viral chest infection for at least two weeks preceding recruitment and any children presenting symptoms of chest infection were excluded. The definition of persistent wheeze was based on physician diagnosis. Subjects were allocated to the persistent wheeze cohort if they had a history of recurrent or persistent wheeze during the preceding 12 months and displayed wheeze in the most recent 3 months. All children in the wheeze cohort were stable at the time of sampling and none had received glucocorticosteroids (inhaled or oral) or β-agonists for at least one month prior to recruitment and airway sampling. Tracheobronchial pAEC were obtained via trans-laryngeal, non-bronchoscopic brushings of children through an endotracheal tube as previously described [7,21,26,27]. The study was approved by Perth Children’s Hospital, St John of God Hospital, Subiaco and The University of Western Australia’s Human Ethics Committees and written consent was obtained from each participant’s legal guardian after being fully informed about the nature and purpose of the study. This study utilized conditional reprogramming for culture establishment and expansion of pAEC from children, as previously described [27,28]. All endpoint experiments were performed using bronchial epithelial growth media (BEGM), as previously described [7,26]. Due to limited cell yields from airway brushing and primary culture expansion potential, different participants’ samples were utilized in downstream experiments (Table S5).

### 2.3. Treatment of pAEC with γ-Secretase Inhibitor (GSI) & Blocking Antibodies

To assess the role of Notch signaling during repair, GSI-mediated global Notch inhibition experiments via DAPT or specific Notch2 and Jagged1 antibody blocking experiments were conducted. Once confluent, cell cultures were starved in BEGM (minus EGF) containing either a concentration range of DAPT (1 nM to 10 μM), Notch2 or Jagged1 blocking antibodies (0.1 and 1 μg/mL) for 24 h prior to in vitro scratch wounding. Culture media were replenished every 48 h due to limited DAPT stability at 37 °C. DAPT experiments were compared to the vehicle control containing equivalent levels of DMSO to the maximal DAPT concentration, that is 0.1% (*v*/*v*) DMSO diluted in BEGM (minus EGF). Blocking antibody-treated cultures were compared to isotype control antibody (IgG1κ, MOPC-21) prepared at the maximum concentration (1 μg/mL). 

### 2.4. Vector Construction 

The MSCV-IRES-GFP plasmid was used as a recipient plasmid following BamHI and NotI restriction digest to excise the IRES-GFP fragment. The Notch2.GFP gene was excised from the donor pcDNA3.1 Notch2.GFP plasmid by HindIII and PspOMI restriction digest. The Notch2 fragment was then gel purified, extracted using QIAquick kit (QIAGEN, Hilden, Germany) and ligated to the MSCV plasmid at a ratio of 3:1 using T4 DNA ligase (Figure S1). The MSCV-Notch2 plasmid was then transformed into 10-beta competent cells and streaked onto lysogeny broth agar (1.6% *w*/*v* agar supplemented with 50 μg/mL carbenicillin). Following overnight incubation at 37 °C, individual colonies were selected and cultured in lysogeny broth supplemented with 50 μg/mL carbenicillin. Plasmids were extracted using Plasmid Plus Maxi Kit (QIAGEN) and the sequence was confirmed by DNA sequencing (Australian Gene Research Facility, Perth, WA, Australia).

### 2.5. Vector Propagation 

The HEK293T cell line was initially expanded, and then 3 × 10^6^ cells were seeded onto a 100 mm dish in complete DMEM media. The following day, cells were transfected with MSCV-Notch2-GFP and the packaging plasmids pCAG-GAG-POL and pCAG-VSVG (kindly provided by Dr Martine Roussel and Dr Arthur Nienhuis) using the reagent FuGene 6 at a ratio of 6:3:1, respectively. On day 4, retroviral particles were collected and stored overnight on ice, and the media were replaced with fresh media. The virus collection was then repeated the following day. Pooled MSCV-Notch2-GFP supernatant was immediately aliquoted and snap-frozen on dry ice before long term storage at −80 °C. Titre was determined by transducing NIH-3T3 fibroblast cells for 72 h and the percentage of GFP expressing cells quantified by flow cytometry (FACS Canto II, BD). Viral titre was determined to be 0.8 × 10^7^/mL for the MSCV-GFP empty vector and 1.2 × 10^7^/mL for MSCV-Notch2-GFP. Flow cytometry data were analysed using FlowJo software v10.4 (FlowJo LLC, Ashland, OR, USA).

### 2.6. Generation of Stable Notch2-GFP-Expressing pAEC Cultures 

Cultured pAEC were seeded into three T25 flasks at 5 × 10^3^ cells/cm^2^ and the following morning, media were refreshed with DMEM alone. MSCV-Notch2-GFP or MSCV-GFP empty vector control was added to a separate flask each at MOI of 5 (or approximately 5 × 10^6^ virions per flask), with the remaining flask serving as an untransduced control. After 3 h, complete growth media were added to flasks and cells were expanded until approximately 80% confluent. Following differential trypsinization, pAEC positive for GFP expression was confirmed (>95%) by flow cytometry (FACS Canto II, BD) and Tali™ Image-Based Cytometer (Thermo Fisher Scientific). Flow cytometry data were analysed using FlowJo software v10.4 (FlowJo LLC). Stably transduced Notch2-GFP pAEC cultures and controls were then further expanded for two additional passages and used for wound repair experiments. 

### 2.7. RNA Extraction and Gene Expression Analysis

Total RNA was extracted using Ambion PureLink RNA mini kit (Thermo Fisher Scientific, Waltham, MA, USA), as previously described [11]. The mRNA expression of Notch receptors, ligands and housekeeping gene, *18S*, were determined using specific primers listed in the Online Supplement (Table S6). Gene expression was determined via two-step reverse transcription and real-time PCR assays and calculated using the 2^−ΔΔCT^ method by normalization to *18S* housekeeping gene and an endogenous tissue control, as previously described [7]. 

### 2.8. In-Cell™ Western Assay

Protein expression of Notch2 and Jagged1 was determined using specific antibodies and quantified via In-Cell™ Western Assay, as previously described [9]. Protein expression specific for Notch2 (1:250, R&D Systems, Minneapolis, MN, USA) and Jagged1 (1:250, R&D Systems) was detected and normalized to cell number using DRAQ5 and Sapphire700 stains. 

### 2.9. Immunocytochemistry and Microscopy

For protein expression and localization analysis of Notch2 in pAEC cultures, fluorescent immunocytochemistry and confocal microscopy were utilized. For imaging of cultured pAEC, cells were cultured on 0.8 cm round glass coverslips (pre-coated with fibronectin, type I collagen and bovine serum albumin) grown to confluence, scratch wounded with a sterile p300 pipette tip and fixed 24 h later fixed using 4% (*w*/*v*) formaldehyde for 20 min at room temperature and washed three times for 5 min per wash with PBS. Cultured pAEC were stained for Notch2-intracellular domain (1:200; Cell Signaling Technologies) and visualized with secondary antibodies Alexa Fluor^®^ 568 goat anti-rabbit IgG (1:200; Molecular Probes, Eugene, OR, USA), as previously described [7]. Fluorescence images were acquired using a Nikon^®^ A1 inverted confocal microscope (Nikon, Minato, Tokyo, Japan), with a Nikon^®^ Plan Apo VC 60x Numerical Aperture (NA) 1.4 oil immersion objective (Nikon) and processed with Nikon Instrument Software (NIS) Elements-Advanced Research (AR) (v4.2.22; Nikon) and ImageJ software. Individual channels were captured sequentially, where a 405 nm laser was used for DAPI with collection through a 450/50 bandpass filter, GFP in retroviral-infected cells excited using a 488 nm laser with collection through 525/50 and AF568 excited with a 561 nm laser and collected through a 585/50 bandpass filter. Z-stack images with a step size of 0.5 µm were collected with a pinhole of 35.8 µm (1.2 AU for 488 nm laser), where the top and bottom of the stacks were determined visually. 

### 2.10. Cell Proliferation Assay

Cell proliferation was assessed using a 3-[4,5-dimethylthiazol-2yl]-5-[3-carboxymethoxyphenyl]-2-[4-sulfophenyl]-2H-tetrazolium inner salt (MTS) assay (Promega; Madison, WI, USA) successfully used on pAEC previously [7,11].

### 2.11. Wound Repair Assays

Two in vitro scratch wound models, routinely performed in our laboratory [10,11,21,29] were utilized. Firstly, to quantify repair kinetics, linear scratch wounds were created using a commercial monolayer wounding kit [11]. Wound closure was calculated through image analysis by the accompanying live-cell imaging system (Essen Bioscience, Ann Arbor, MI, USA). Secondly, to quantify gene expression perturbations post-wounding, scratch wounds were made using a plastic p300 pipette tip as described [10]. 

### 2.12. Interrogation of Publicly Available Bulk and Single-Cell Transcriptomic Datasets

Two published bulk transcriptomic datasets available in the National Center for Biotechnology Information Gene Expression Omnibus, accession IDs GSE118761 [30] and GSE145505 [31] were selected based on utilizing comparable ex vivo pAEC isolation protocols, that is, nasal and lower airway samples from children with and without wheeze. These studies used comparable transcriptomic platforms, that is, Illumina^®^ HiSeq 2500 50-bp single-end reads (v4 chemistry) and data analysis pipelines. Differentially expressed gene lists comparing gene expression in ex vivo pAEC from children with and without wheeze were investigated. Using the prior knowledge of protein:protein interactions, minimum first-order networks were generated with NetworkAnalyst, and pathway analysis (reactome) was performed where datasets were screened for an overrepresentation of the Notch signaling pathway [32]. Furthermore, to determine the cell-specific expression and biological relevance of Notch receptors and ligands of interest in the airway epithelium, a curated set of publicly available human airway epithelial single-cell RNA-Seq (scRNA-Seq) datasets in the GEO repository (accession ID GSE102580) was interrogated [33]. This dataset measured the transcriptomes of airway epithelial cell populations from human donors (*n* = 3; 2970 cells) and mice (*n* = 4; 7662 cells), with varying numbers of cells. Data are presented with SPRING plots using a graph-based algorithm that conserves neighboring relationships of gene expression in airway epithelial cells across all differentiation trajectories [34].

### 2.13. Statistics

Statistical significance (* *p*-value < 0.05) for comparisons between two groups was determined by Fisher’s exact test for categorical variables or the Mann–Whitney U-test for continuous variables, and Kruskal–Wallis test with Dunn’s multiple comparisons testing for more than two groups’ comparisons. Data are presented as median with interquartile range (IQR; 25% percentile-75% percentile), unless stated otherwise. All experiments were performed in duplicate using at least five individual patients of each cohort per experiment, or as otherwise stated.

## 3. Results

### 3.1. Identification of Notch Signaling Pathway in Pediatric Respiratory Wheeze Cohorts

**Figure 1:**
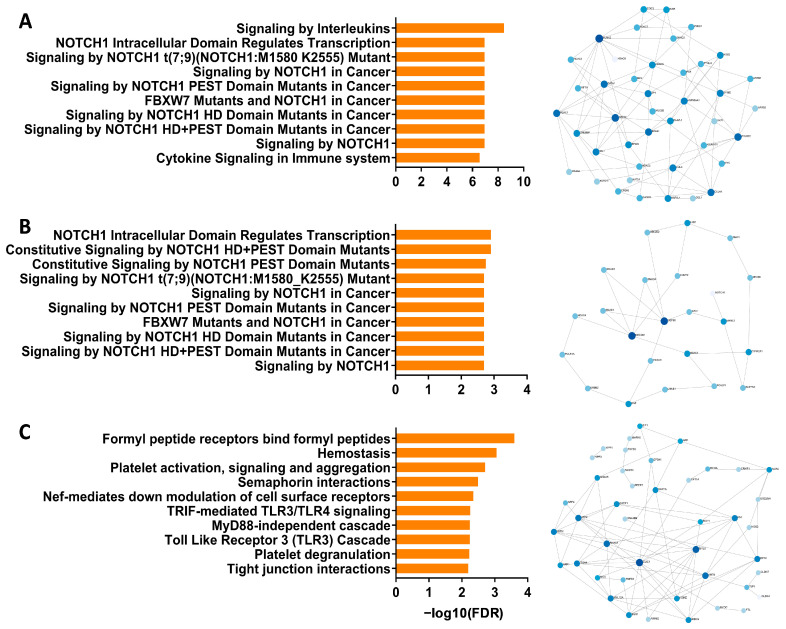
Interrogation of the Notch signaling pathway in published airway epithelial RNA-Seq datasets from children with wheeze. (**A**) Top 10 pathways (reactome) enriched in differentially expressed genes from ex vivo lower airway epithelial samples from children with recurrent wheeze compared to non-wheeze controls. Minimum network map of genes associated with the Notch signaling pathway (reactome) in lower airway epithelial samples from children with recurrent wheeze. (**B**) Top 10 pathways (reactome) enriched in the M14 module associated with Injury Response in ex vivo nasal epithelial samples from an independent cohort of children with high wheeze and low lung function. Minimum network map of genes associated with the Notch signaling pathway (reactome) in the M14 module. (**C**) Top 10 pathways (reactome) enriched in the M20 module associated with Epithelial Integrity in ex vivo nasal epithelial samples from children with high wheeze and low lung function. Minimum network map of genes in the M20 module where there was a lack of association with the Notch signaling pathway. The size of nodes indicates a larger number of gene:gene connections, or high hub degree, and color saturation indicates their betweenness, or centrality value, within the network.

To address whether Notch signaling is dysregulated in airway epithelial samples from children with wheeze, we initially performed a meta-analysis of relevant pediatric-specific published transcriptomic datasets [30,31] to identify any association of Notch signaling with recurrent wheeze, and/or epithelial injury and repair. Specifically, we targeted our comparisons to an ex vivo pediatric lower airway transcriptomic dataset collected independently by our team [30], as well as an ex vivo pediatric nasal transcriptomic dataset that identified two gene modules associated with ‘injury response’ and ’epithelial integrity’ [31]. Pathway analysis performed on the differentially expressed genes in lower airway epithelial samples from children with and without wheeze [30] identified Notch signaling as well as downstream Notch effector gene transcription being the most over-represented pathways (Figure 1A). It also revealed key Notch transcription co-activators (KAT2A, CREBBP, EP300), co-repressors (HDAC1-3, 5, 8), downstream target genes (e.g., MYC) and potential inhibitors of Notch signaling (ARRB1-2) also being differentially expressed (Figure 1A). 

We then went onto interrogating two gene modules from an independent pediatric wheeze cohort [31] that was associated with frequent wheezing episodes and epithelial vulnerabilities (i.e., M14: ‘injury response’ and M20: ‘epithelial integrity’). Significantly, pathway analysis of the M14 module corroborated observations made using the first dataset, identifying Notch signaling and downstream Notch effector gene transcription as being the most over-represented pathways (Figure 1B). Network analysis highlighted central Notch pathway genes, including upstream regulators of Notch (MECOM, USP9X), NOTCH1 receptor, as well as Notch co-repressors (HDAC1, 4, 6) (Figure 1B). Importantly, the second gene module, M20, that was associated with frequent wheeze and epithelial integrity in the independent dataset did not associate with Notch signaling (Figure 1C). In fact, pathways and key network hubs associated with known epithelial repair mechanisms, including cell–cell junctions and extracellular interactions (Figure 1C). With meta-analysis from independently curated datasets supporting our hypothesis that dysregulated Notch signaling contributes to the defective airway epithelial repair in children with wheeze, we then went onto establish expression profiles of Notch receptors and associated ligands on primary airway epithelial cells derived from children with and without wheeze. 

### 3.2. Altered Expression Profiles of Notch Receptors and Ligands in Ex Vivo pAEC from Children

**Figure 2:**
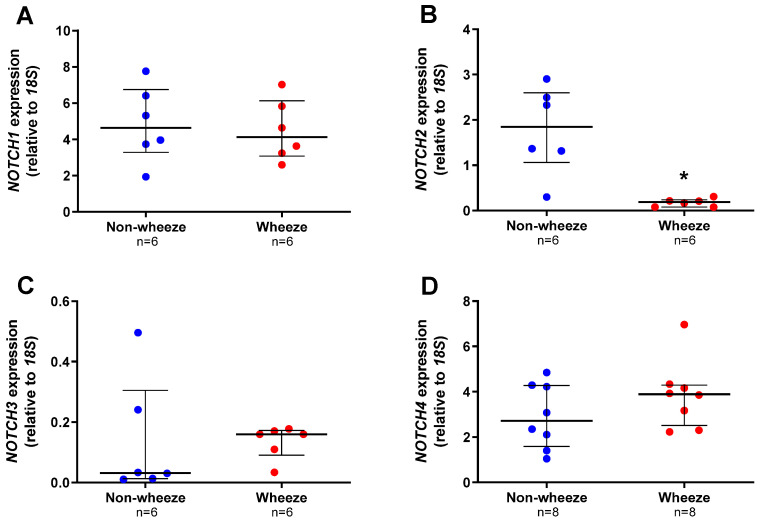
Notch receptors gene expression profiles in ex vivo pAEC from children with and without wheeze. Relative expression of NOTCH1–4 receptors (**A**–**D**) were measured in ex vivo pAEC from children with and without wheeze (*n* ≥ 6) by real-time qPCR (refer to 2.5.4). (**A**) NOTCH1 was the most abundantly expressed Notch receptor gene in ex vivo pAEC from a paediatric cohort. However, NOTCH1 mRNA expression was not significantly different between pAEC from children without wheeze. (**B**) In contrast, mRNA expression of NOTCH2 was significantly lower in pAEC from children with wheeze compared to their non-wheeze counterparts. (**C**,**D**) Finally, no significant differences were observed between pAEC from children with and without wheeze in mRNA expression of NOTCH3 and NOTCH4, although the genes were expressed at different magnitudes. Each dot represents the mean of two replicates from a single ex vivo sample. Data are normalized to housekeeping gene, 18S and represented as median ± IQR. * Statistical significance relative to the non-wheeze cohort (*p* < 0.050; Mann–Whitney U-test).

All Notch receptors were found to be expressed by pAEC from children without wheeze, although at different magnitudes (median, IQR; *NOTCH1*: 4.638, 3.290–6.748; *NOTCH2*: 1.847, 1.061–2.599; *NOTCH3*: 0.032, 0.013–0.305, and *NOTCH4*: 2.715, 1.581–4.274 AU; Figure 2A–D). Notch receptors were also expressed in pAEC from children with wheeze (*NOTCH1*: 4.133, 3.076–6.129; *NOTCH2*: 0.188, 0.0784–0.239; *NOTCH3*: 0.160, 0.091–0.173, and *NOTCH4*: 3.893, 2.519–4.292 AU; Figure 2A–D). Significantly, the mRNA expression of only one Notch receptor, *NOTCH2*, was significantly lower in ex vivo pAEC from children with wheeze (10-fold, *p* = 0.004, Mann-Whitney U-test; Figure 2B) compared to non-wheeze controls. 

**Figure 3:**
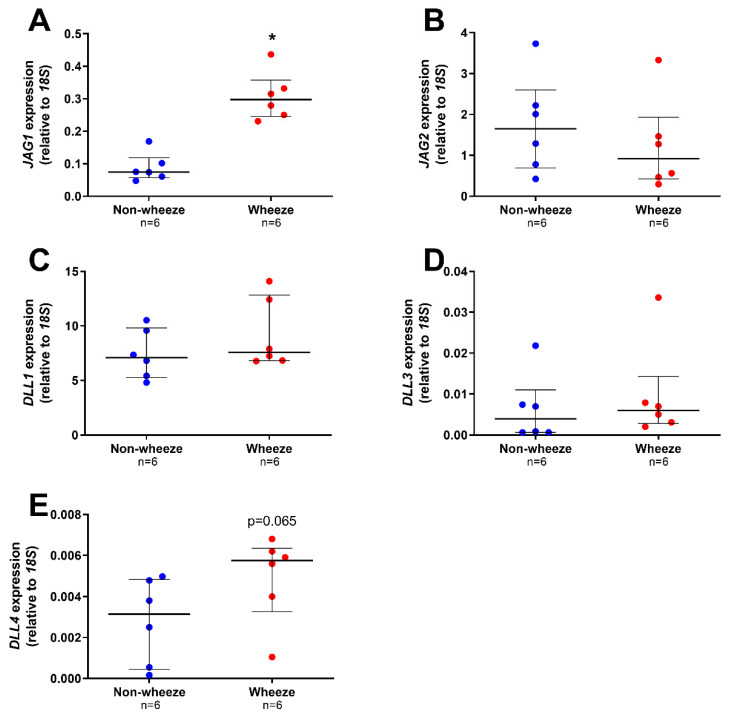
Notch ligands gene expression profiles in ex vivo pAEC from children with and without wheeze. Relative expression of Notch ligands; JAG1–2 (**A**,**B**) and DLL1-4 (**C**,**E**) were measured in ex vivo pAEC from children with and without wheeze (*n* = 6) by real-time qPCR. (**A**) mRNA expression of JAG1 was significantly increased in pAEC from children with wheeze compared to their non-wheeze counterparts. (**B**) JAG2 mRNA expression was not significantly different between pAEC from children with and without wheeze. (**C**,**E**) DLL1 was the most abundantly expressed Notch ligand in ex vivo pAEC from a paediatric cohort. However, no significant differences were observed between pAEC from children with and without wheeze in mRNA expression of DLL1 (**C**), DLL3 (**D**) and DLL4 (**E**), although the genes were expressed at different magnitudes. Each dot represents the mean of two replicates from a single ex vivo sample. Data are normalized to housekeeping gene, 18S and represented as median ± IQR. * Statistical significance relative to the non-wheeze cohort (*p* < 0.050; Mann–Whitney U-test).

All the Notch ligands were found to be expressed in pAEC from children without wheeze, although at different magnitudes (median, IQR; *JAG1*: 0.075, 0.057–0.119; *JAG2*: 1.649, 0.690–2.598; *DLL1*: 7.076, 5.263–9.805; *DLL3*: 0.004, 0.001–0.011, and *DLL4*: 0.003, 0.001–0.005 AU; Figure 3A–E). Notch ligand gene expression was also present in pAEC of children with wheeze (median, IQR; *JAG1*: 0.297, 0.246–0.358; *JAG2*: 0.919, 0.423–1.931; *DLL1*: 7.568, 6.802–12.834; *DLL3*: 0.006, 0.003–0.014, and *DLL4*: 0.006, 0.003–0.006 AU; Figure 3A–E). However, only one Notch ligand, *JAG1*, was significantly higher in ex vivo pAEC from children with wheeze (3.9-fold, *p* = 0.002, Mann-Whitney U-test; Figure 3A) compared to non-wheeze controls. 

Notch pathway gene expression profiles were ascertained from ex vivo airway brushings containing mixed airway epithelial cell subsets (e.g., ciliated, secretory and basal). To determine the contribution of different airway epithelial cell subsets to the Notch pathway gene expression profiles, publicly available single-cell RNA-Sequencing data from mouse (Figure S3A) and human (Figure S3B) tracheobronchial epithelial cells were mined (refer to Online Supplement). The proportion of airway epithelial cell subsets in these datasets were identified to be primarily basal, secretory, ciliated and intermediate cell types (e.g., basal > secretory), as well as rare cell types (Figure S2). Significantly, *NOTCH2*, *JAG1* and Notch signaling downstream target, *HES1* gene expression, were all found to be predominantly expressed by airway progenitors, i.e., basal and secretory cells (Figure S3). Considering our previous findings of a dysregulated reparative capacity by airway basal epithelial cells [10,11,21], we thus interrogated the role of Notch signaling in this larger airway progenitor cell subset.

### 3.3. Dysregulated Expression of NOTCH2 and JAG1 during In Vitro Wound Repair in pAEC Cultures from Children

**Figure 4:**
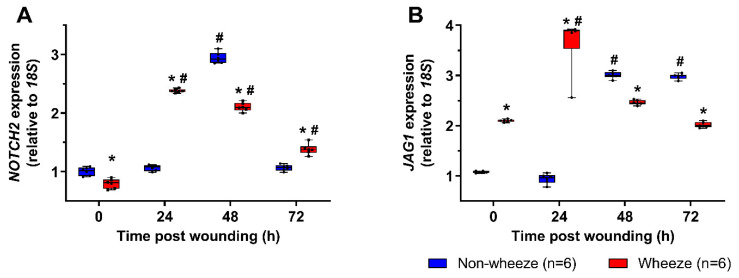
*NOTCH2* and *JAG1* gene expression profiles in pAEC post in vitro wounding. Relative expression of *NOTCH2* (**A**) and *JAG1* (**B**) mRNA were measured in pAEC post in vitro wounding from children with (*n* = 6) and without wheeze (*n* = 6) by real-time qPCR. (**A**,**B**) Following wounding, pAEC from children without wheeze displayed a significant upregulation of *NOTCH2* and *JAG1* mRNA expression at 48 h post wounding respectively. By 72 h post wounding, *NOTCH2* mRNA expression (**A**) had returned to baseline levels, whilst *JAG1* mRNA expression (**B**) was maintained at about 3-fold higher levels than baseline. In contrast, pAEC cultures from children with wheeze displayed lower gene expression levels of *NOTCH2* (A) and higher gene expression levels of *JAG1* (**B**) at baseline. An earlier induction of *NOTCH2* mRNA expression was observed in pAEC from children with wheeze at 24 h post wounding, which was sustained to 72 h post wounding. Although *JAG1* mRNA levels were induced by 24 h post wounding, *JAG1* expression returned to baseline levels by 72 h post wounding indicating distinct differences in the kinetics of Notch signaling during airway epithelial repair in children with wheeze. Data are represented as median ± IQR. * Statistical significance of the wheeze group relative to matching non-wheeze timepoint sample (*p* < 0.050; Mann–Whitney U-test), or # statistical significance of relevant timepoint to time 0 h of matching patient cohort (*p* < 0.050; Kruskal–Wallis test with Dunn’s test for multiple comparisons).

Since dysregulated expression of *NOTCH2* receptor and its ligand, *JAG1,* were observed in ex vivo airway brushings of children with wheeze, the kinetics of *NOTCH2* and *JAG1* mRNA were investigated following in vitro wounding of pAEC cultures. Post wounding, pAEC from children without wheeze displayed a significant upregulation of *NOTCH2* (3-fold, *p* < 0.050; Figure 4A) and *JAG1* (3-fold, *p* < 0.050; Figure 4B) at 48 h post wounding compared to time 0 h. By 72 h post wounding, *NOTCH2* mRNA expression had returned to baseline levels (*p* = 0.104; Figure 4A) whilst *JAG1* mRNA expression was maintained at higher levels (3-fold, *p* < 0.050; Figure 4B) compared to time 0 h. 

*NOTCH2* mRNA levels observed at time 0 h were reduced (1.3-fold *p* < 0.050) in pAEC from children with wheeze compared to non-wheeze children (Figure 4A). Furthermore, pAEC isolated from children with wheeze displayed 2-fold higher mRNA levels of *JAG1* (*p* < 0.050, Figure 4B) at baseline compared to non-wheeze controls, mirroring the ex vivo data (Figure 4B). Cell cultures from children with wheeze had higher *NOTCH2* (2.5-fold, *p* < 0.050) and *JAG1* (4-fold, *p* < 0.050) mRNA expression kinetics post-wounding with an earlier induction of both genes at 24 h compared to their non-wheeze counterparts. Unlike the non-wheeze controls, increased gene expression of *NOTCH2* persisted in cultures from children with wheeze at 48 h (2-fold, *p* < 0.050) and 72 h (1.3-fold, *p* < 0.050) post-wounding compared to baseline. Conversely, *JAG1* mRNA expression returned to baseline levels after 48 h and 72 h (*p* > 0.050) post-wounding, unlike the sustained increased expression observed in pAEC from children without wheeze (Figure 4B).

### 3.4. Inhibition of Notch Signaling Abrogates pAEC Wound Repair In Vitro

**Figure 5:**
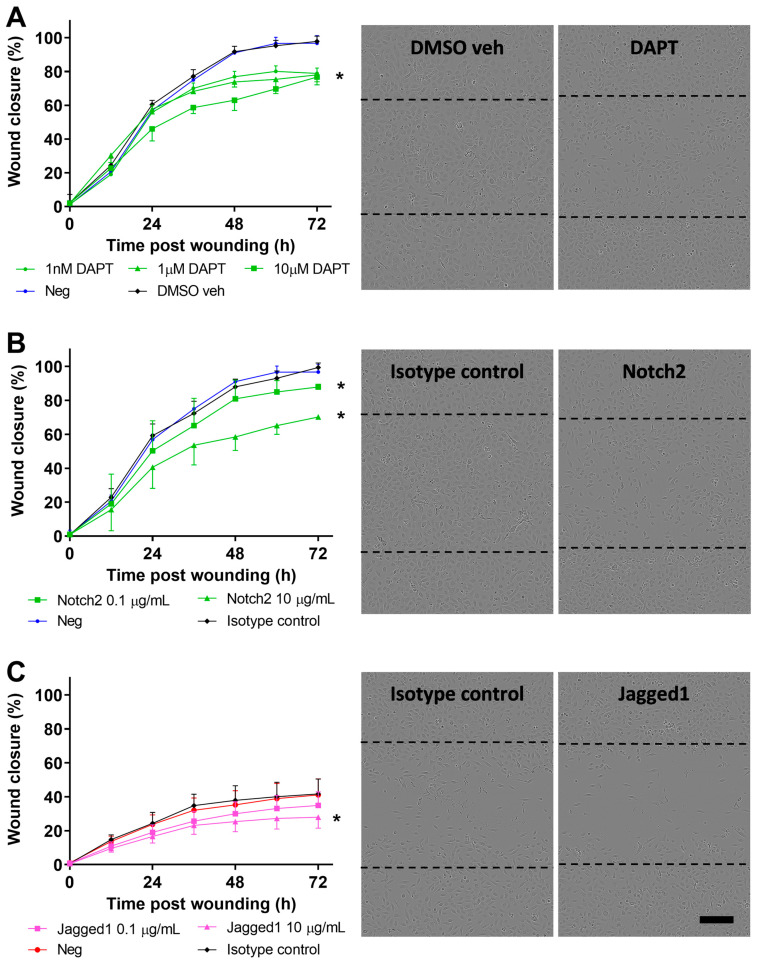
Blocking of Notch signaling in pAEC of children without wheeze. (**A**) Monolayer pAEC cultures from children without wheeze were treated with increasing concentrations (1 nM–10 μM; green circles, triangles, squares) of the GSI, DAPT 24 h prior to wounding. Wound closure rates of pAEC from children without wheeze treated with DAPT, DMSO vehicle (black diamonds) (0.1% *v*/*v*) or untreated negative control (blue circles) were determined. Full wound closure in DMSO vehicle and untreated negative controls were achieved by approximately 54 h post-wounding. Cultures treated with DAPT showed a concentration-dependent reduction in wound closure and full wound closure was not achieved within the completion of the experiment at 72 h post-wounding. Representative phase-contrast images of DMSO vehicle control (0.1% *v*/*v*) and DAPT (10 µM) treated cultures from children without wheeze at 72 h post-wounding. (**B**) Monolayer pAEC cultures from children without wheeze were treated with either 0.1 (green squares) or 10 (green triangles) µg/mL Notch2 blocking antibody 24 h prior to and during wounding. Notch2 blocking (10 µg/mL; green triangles) resulted in reduced wound closure compared to the negative and isotype antibody controls (10 µg/mL). Representative phase-contrast images of cultures from children without wheeze treated with either isotype antibody control or Notch2 antibody (10 µg/mL) at 72 h post wounding. (**C**) Monolayer pAEC cultures from children with wheeze were treated with either 0.1 (purple squares) or 10 (purple triangles) µg/mL Jagged1 blocking antibody 24 h prior to and during wounding. Jagged1 blocking (10 µg/mL) resulted in a marginal reduction in wound closure compared to the negative and isotype antibody controls (10 µg/mL). Representative phase-contrast images of cultures from children without wheeze treated with either isotype antibody control or Jagged1 antibody (10 µg/mL) at 72 h post wounding. Experiments were performed in cultures from children with (*n* = 6) or without wheeze (*n* = 5). Data are represented as mean ± SD. * Statistical significance relative to DMSO vehicle or isotype antibody controls (*p* < 0.050, Friedman test with Dunn’s multiple comparisons test). Dashed lines in phase-contrast images indicate the original wound area at t = 0 h post-wounding. Scale bar is 200 µm (10× objective).

Broad modulation of the Notch signaling pathway was attempted by inhibiting NICD cleavage and signal transduction using the GSI, DAPT. Specifically, untreated or DMSO vehicle (0.1% *v*/*v*) treated negative control pAEC cultures from children without wheeze reached full wound closure by approximately 54 h post-wounding (Figure 5A). However, cultures treated with various concentrations of DAPT (1 nM to 10 µM) failed to achieve full wound closure within the completion of the experiment at 72 h post-wounding (Figure 5A). Although there was varied wound closure responses by DAPT-treated cells, there was a consistently significant inhibition of wound closure post-Notch signaling inhibition with all tested concentrations of DAPT (*p* < 0.050, Figure 5A).

Blocking of Notch2 with a monoclonal antibody (0.1 and 10 µg/mL) in pAEC cultures from children without wheeze resulted in significantly reduced wound closure rates compared to negative isotype antibody control (*p* < 0.050, Figure 5B). Conversely, specific antibody blocking (10 µg/mL) of Jagged1 in pAEC cultures from children with wheeze showed a marginal reduction in wound closure rates compared to the negative isotype antibody control (*p* < 0.050, Figure 5C).

### 3.5. Overexpression of Notch2 Has no Effect on the Reparative Capacity of pAEC from Children with Wheeze

**Figure 6:**
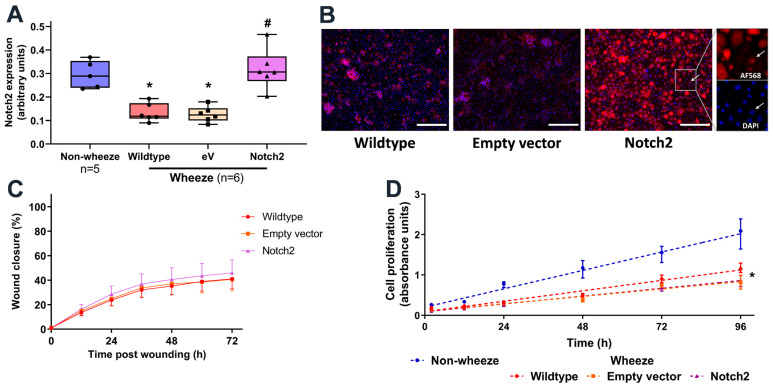
Overexpression of Notch2 in pAEC from children with wheeze. (**A**–**D**) Cultures from children with wheeze were infected with a retrovirus containing either the empty vector-GFP or Notch2-GFP. (**A**) Protein expression of Notch2 was measured by In-Cell Western and normalized to cell number. (**B**) Immunofluorescent staining for Notch2 intracellular domain protein identified its nuclear staining in Notch2-overexpressed cultures. Scale bar: 200 µm (20× magnification) (**C**) Monolayer pAEC cultures expressing Notch2-GFP (purple triangles) or empty vector-GFP (orange squares) and wildtype controls (red circles) displayed comparable repair capacity following in vitro scratch wounding. (**D**) Cultures from children with wheeze (red circles) showed significantly lower cell proliferation rates compared to non-wheeze controls (blue circles). Notch2 overexpression (purple triangles) had no effect on cell proliferation compared to empty vector-GFP (orange squares) or wildtype wheeze controls (red circles). Experiments utilized pAEC cultures from children with (*n* = 6) and without wheeze (*n* = 5). Data are represented as median ± IQR (* *p* < 0.050, wheeze vs. non-wheeze; # *p* < 0.050, empty vector/Notch2 vs. Wildtype control; Dunn’s multiple comparisons test).

Next, we evaluated the role of Notch2 overexpression using a retroviral system in submerged monolayer pAEC cultures from children with wheeze. Successful retrovirus infection efficacy in primary cultures was confirmed as GFP positive cells by flow cytometry (Figure S4A) and epifluorescence microscopy (Figure S4B). Additionally, overexpression of Notch2 was validated at the protein level, where overexpressed levels in pAEC cultures from children with wheeze were comparable to those observed in cultures from children without wheeze (Figure 6A). Notch2 overexpression resulted in hyperactivated Notch2 signaling as demonstrated by increased nuclear staining in transduced cultures (Figure 6B). 

To determine whether overexpression of Notch2 expression is sufficient to rescue the defective airway epithelial cell repair phenotype in children with wheeze, in vitro wound repair capacity was assessed in pAEC cultures either untreated or infected with empty vector or Notch2 retrovirus (Figure 6C). Overexpression of Notch2 showed no significant effect on the pAEC reparative capacity (Figure 6C). In addition, although cultures from children with wheeze had a slower proliferation rate compared to children without wheeze, Notch2 overexpression did not modulate cell proliferation (Figure 6D).

## 4. Discussion

This study provides new insights into the mechanisms regulating airway epithelial repair in the context of childhood wheeze. In the present study, a meta-analysis of published transcriptomic datasets identified dysregulated Notch signaling to associate with epithelial repair and integrity in upper and lower airway samples from children with recurrent wheeze. The expression profile of Notch receptors and ligands was investigated in pAEC cultures both at basal levels and in response to wounding. The data indicate that, at baseline, pAEC of children with and without wheeze expressed all Notch receptors and ligands, although at different magnitudes. In particular, *NOTCH2* and *JAG1* were found to have significantly lower and higher mRNA expression levels, respectively, in both ex vivo and in vitro pAEC from children with wheeze. Following wounding, pAEC from children with wheeze also showed altered gene expression kinetics of *NOTCH2* and *JAG1* as compared to pAEC from children without wheeze. Furthermore, inhibition of Notch signaling in pAEC from children with and without wheeze partially impaired wound closure rates in vitro. Finally, overexpression of Notch2 failed to modulate the reparative and proliferative capacity of pAEC cultures from children with wheeze. Collectively, the data generated in this study provide evidence of imbalanced Notch signaling partially contributing to dysregulated airway epithelial cell repair in cultures from children with wheeze. Our data suggest that Notch pathway inhibition may partially abrogate the reparative capacity of airway epithelial progenitor cells. Further studies are required to elucidate the functional role of Notch in airway epithelial cell repair following in vivo injury, as well as the safety and efficacy of Notch-targeting therapeutics in chronic obstructive airway diseases such as chronic asthma. 

The present study profiled the gene expression of all Notch receptors and ligands and determined that pAEC expressed *NOTCH1* receptor most abundantly compared to *NOTCH2*–*4*. Furthermore, *JAG2* and *DLL1* were the highest expressing Notch ligands in pAEC from children. To date, there have been no other studies profiling the Notch pathway in detail from airway samples of children with wheeze or healthy controls. Some studies have investigated the transcriptional expression profiles of Notch receptors and ligands in murine endothelial and melanoma cell lines [35] and basal epithelial and differentiated epithelial cells isolated from steady-state adult mouse tracheobronchial linings [36]. Similar to the findings of this study, murine basal airway epithelial cells expressed Notch1 as the predominant Notch receptor, although Notch-2 and -3 were also detected at lower magnitudes of expression [36]. Furthermore, Jagged-2 and DLL1 were the predominant Notch ligands in murine basal epithelial cells [36]. These expression pattern similarities in Notch receptors and ligands between human and murine airway epithelia could suggest that investigations of the Notch signaling pathway in murine models could be complementary to human airways, particularly for gene modulation investigations. Through the mining of single-cell RNA-Sequencing datasets, our study identified differential expressions of Notch pathway genes to airway epithelial progenitors, such as basal and secretory cells. However, these analyses were not confirmed at the protein level in airway samples from children. Future studies could investigate the expression patterns of Notch receptors and ligands in differentiated cells, as reported by Rock and colleagues [36], to eliminate the potential variability based on epithelial cell heterogeneity. 

Interrogation of the gene expression of Notch receptors and ligands in ex vivo pAEC from children with wheeze identified that *NOTCH2* is decreased and *JAG1* increased, compared to their non-wheezing counterparts. Importantly, these observations were confirmed in submerged basal epithelial cell monolayer cultures. Collectively, the findings of the present study support the hypothesis that the Notch pathway is dysregulated in the airways of children with wheeze. Previous investigations have identified the Notch signaling pathway as a regulator of migration in neural stem cell niches [37] as well as surface epithelia including airways, cornea and skin [18,38,39]. In addition, several studies using murine models of allergic airways disease have identified the role of Notch signaling on regulating immune cell function known to impact airway repair [20,40,41,42,43,44,45]. However, the role of the Notch signaling pathway in airway epithelial repair and its regulation of potential cross-talk between immune cells in the airway remains poorly understood. Our study adds to previous investigations of the Notch pathway dysregulation in adult asthma and expands these findings to early childhood wheeze, highlighting its potential as a target for therapy in early life. 

In the present study, the gene expression kinetics of *NOTCH2* and *JAG1* were measured following injury to better understand their role during the repair. *NOTCH2* and *JAG1* were significantly upregulated at 48 h post wounding; however, there was an earlier induction of both genes observed in pAEC from children with wheeze by 24 h post wounding. These data suggest that imbalanced regulation of the Notch signaling pathway components could impair airway epithelial cell repair of the airway epithelium of children with wheeze. Of note, Jagged1-dependent activation of Notch signaling was reported to impede on cell migration and repair by strengthening integrin-mediated adhesion to the matrix [16,37,46,47]. Furthermore, upon receptor activation, Notch2 has been suggested to hyper-phosphorylate, leading to NICD nuclear translocation and transactivation of its target gene, *HES1*, transcription in the nucleus [48]. However, as downstream Notch signals are only transmitted through receptor cleavage [49], Notch signaling may be abrogated due to reduced levels of Notch2 receptor in pAEC from children with wheeze. This study did not investigate the activation state of Notch signaling or the cellular levels of NICD and downstream targets like *HES1* during a physiological repair response or in disease. Of note, the meta-analysis of the published transcriptomics datasets highlighted potential dysregulation of upstream regulators able to activate (MECOM and USP9X) [50,51] or inhibit Notch (e.g., ARRB1) [52]. In addition, this meta-analysis indicated additional complexity in Notch regulation through potential alteration of Notch co-activators (KAT2A, CREBBP, EP300) and co-repressors (HDACs) that modulate its downstream target gene transcription and cellular functions [23]. Future studies could elucidate whether regulatory mechanisms of Notch contribute to defective airway epithelial repair in the airways of children with wheeze.

Next, global inhibition of Notch signaling using DAPT treatment in pAEC cultures from children without wheeze was utilized to examine the role of the pathway independently of potential receptor-ligand interactions. As hypothesized, the inhibition of Notch signaling was found to abrogate repair, although only partially. Similarly, specific inhibition of Notch2 and Jagged1 with blocking antibodies resulted in a marginal reduction in pAEC wound closure. These observations contrast with findings in the current literature that suggest inhibition of Notch signaling accelerates repair in murine and human corneal or adenocarcinoma mammary epithelial cells [17,18,39]. These different observations may be attributed to differences between the type of epithelia and human cohort sources, such as pAEC cultures from adults versus children as utilized in the present study. Cell and tissue specific differences have been observed in Notch signaling due to the dynamic equilibrium of Notch receptors and ligands and their interaction with their respective microenvironment [46,53]. Importantly, the Notch signaling pathway has been found to partially regulate repair processes via cross-talk with other signaling pathways like PI3K/Akt and regulating integrin expression and activity, and vice versa where integrins are necessary for appropriate upregulation of Notch signaling [16,18,37,39,47,54,55]. Our meta-analysis of the epithelial repair-associated gene modules in the study by Altman et al. [31] highlighted the role of cell–cell and cell–extracellular interactions (M20), in addition to Notch signaling (M14). It is likely that additional mechanisms are at play that results in aberrant epithelial repair. For example, the role of tumour protein (TP)-63, basal airway epithelial cell master-regulator, has been implicated in epithelial progenitor cell fate determination and repair processes through the regulation of Jagged-1 expression and the Notch signaling pathway [38,56]. In fact, our team and others have previously demonstrated [11,56,57] that pAEC from children and adults with wheeze or asthma have reduced integrin expression and downstream signaling impacting their capacity to repair. 

Inhibition of the Notch pathway alone did not completely replicate the dysregulated epithelial repair observed in pAEC of children with wheeze. Furthermore, in vitro overexpression of Notch2 in pAEC from children with wheeze did not have a detrimental effect on airway epithelial cells; however, it failed to rescue the defective airway epithelial cell repair. The interrogation of publicly available single-cell transcriptomics datasets in this study identified *NOTCH2*, *JAG1* and downstream effector (*HES1*) gene expression in basal and secretory/ciliated cell subsets. It is possible that aberrant Notch signaling has cell-specific effects on differentiated airway epithelial cells, where Notch2 overexpression may not alter airway basal epithelial cell proliferation and reparative capacity. Importantly, the development of Notch inhibitors as potential therapeutic candidates in severe asthma stems from evidence showing global gamma-secretase inhibitors to reduce Th2 inflammation and mucus hyperproduction in in vitro studies of adult asthma and allergic airways disease in in vivo murine models [19,20,41,45]. Although there is a strong interest in therapeutically targeting Notch signaling in adult patients with asthma, there remains a paucity of data on the role of this pathway in airway progenitor cells during airway epithelial cell repair, which would be integral for early life intervention in children with dysfunctional epithelial repair. Although this study focused on children with persistent wheeze, epithelial targeted treatments in early life are likely to halt symptoms progression and reduce chronic disease burden. There is a need for longitudinal studies assessing the role of Notch across early life and adulthood in airway diseases like persistent wheezing and asthma, to fully characterize the epithelial endotype.

It is appreciated that the Notch signaling pathway would also contribute to the later phases of airway epithelial repair via ciliated and goblet cell differentiation [36,41,58,59]. Importantly, the findings of this study indicating imbalanced Notch signaling in the airways of children with wheeze could potentially contribute to goblet cell hyperplasia and loss of ciliated cells. As a first step, this study was limited to assessing the role of the Notch pathway following injury in airway epithelial progenitor cells (i.e., basal), and as such, the contribution of dysregulated Notch signaling to epithelial differentiation in cultures from wheeze was not investigated. Intrinsic abnormalities of airway progenitors may be confounded by a number of cellular processes, thus, in this study, monolayer basal cell cultures were first used to elucidate the contribution of the Notch pathway in basal cell renewal and repair. The role of the Notch signaling pathway in airway epithelial cell differentiation following damage was beyond the scope of this study; however, future studies may utilize differentiated ALI cultures to better understand these mechanisms in the airways of children with preschool wheeze or school-aged asthma. Although it was not the focus of this study, future research could interrogate the role of the Notch pathway and sex differences in children and adults with asthma, which may affect responses to treatment requiring a precision medicine approach. Previous findings have identified defective airway epithelial cell repair in children with wheeze and asthma to be independent of age and sex [11]. However, therapeutic modulation of Notch may differentially impact other disease factors such as immune skewing in males and females. Our study highlights the need for further studies to determine the therapeutic efficacy of targeting the Notch pathway, particularly on the effects of potential therapeutics on the reparative capability of the airway epithelium. 

In summary, this study is the first to implicate dysregulated Notch signaling in paediatric cohorts, and to report detailed profiling of Notch receptors and ligands in a cohort of young children with or without wheeze. Although the magnitude of Notch receptors and ligands mRNA expression varied, *NOTCH2* and *JAG1* were differentially expressed in pAEC from children with wheeze compared to their non-wheezing counterparts. Furthermore, global and targeted inhibition of Notch signaling leads to a reduction in wound closure rates in pAEC cultures from children with and without wheeze. However, overexpression of Notch2 did not modulate in vitro wound repair capacity in pAEC cultures from children with wheeze. As hypothesized, these findings suggest that dysregulated expression of the Notch signaling pathway’s components partially contribute to defective wound repair of airway epithelial cells from children with wheeze. However, the Notch signaling pathway’s dysregulation alone does not completely account for the defective repair phenotype observed. Similar to findings from previous studies [46,60,61], molecular redundancy and signaling cross-talk within cells and amongst different cell and tissue types exist physiologically and during disease processes. For heterogeneous chronic airway diseases like asthma, it is likely that a combination or system of abnormalities exists contributing to dysregulated repair and barrier restitution following injury. Targeting only Notch signaling may negatively impact the repair of damaged airways, although further research is necessary before commencing clinical assessments of Notch-targeting therapeutics. Collectively, this investigation has provided new insight regarding an important intrinsic epithelial vulnerability in wheeze and has provided a further rationale for investigating epithelium-centred asthma therapies in young children with wheeze or confirmed asthma diagnosis. The development of novel therapeutics targeting the Notch pathway for airway diseases should also investigate effects on airway progenitor cells to account for the potential negative impact on long term airway tissue maintenance and reparative capacity.

## Data Availability

The data presented in this study are available on request from the corresponding author. The data are not publicly available due to ethical restrictions.

## Supplementary Materials

The following are available online at https://www.mdpi.com/article/10.3390/jpm11121323/s1, Figure S1: Preparation of MSCV plasmid for Notch2 overexpression studies, Figure S2: Cellular composition of airway epithelium, Figure S3: Notch gene expression profiles in airway epithelial cell subsets, Figure S4: Confirmation of retroviral infection and integration of MSCV-Notch2-GFP or empty vector MSCV-GFP in pAEC from children with wheeze, Table S1: Reactome pathways associated with differentially expressed genes from lower airway epithelial samples from children with wheeze, Table S2: Reactome pathways associated with differentially expressed genes from lower airway epithelial samples from children with wheeze, Table S3: Reactome pathways associated with differentially expressed genes from lower airway epithelial samples from children with wheeze, Table S4: Study participants demographics, Table S5: Description of study participants demographics for all figures, Table S6: Details of reference and target genes.

## Appendix A

The members of WAERP are: Anthony Kicic, Stephen M. Stick, Elizabeth Kicic-Starcevich, Amy Greenly, Angela Fuery, Luke W. Garratt, Erika N. Sutanto, Kevin Looi, Jessica Hillas, Thomas Iosifidis, Nicole C. Shaw, Samuel T. Montgomery, Kak-Ming Ling, Kelly M. Martinovich, Samantha McLean, Craig Schofield, Renee Ng, Andrew Vaitekenas, Daniel Laucirica, Matthew W-P Poh, Joshua Iszatt, Reanne Ho, Shyan Vijayasekaran, Paul Swan, Mairead Heaney, Ian Forsyth, Tom Rawlings, Neil Chambers, Alexander Larcombe, Francis Lannigan, Riccardo Bergesio, Bernard Lee, Paul Swan, Kate McGee, Nyssa Millington, Rael Rivers, Eugene Roscioli, Timothy Barnett, Cindy Richards, Ee-Lyn Tan, Marina Stubbs. The Australian Respiratory Epithelium Consortium (AusREC) acknowledges the following members: Anthony Kicic, Stephen M. Stick, Elizabeth Kicic-Starcevich, Luke W. Garratt, Erika N. Sutanto, Kevin Looi, Jessica Hillas, Thomas Iosifidis, Nicole C. Shaw, Samuel T. Montgomery, Kak-Ming Ling, Kelly M. Martinovich, Matthew W-P Poh, Daniel R. Laucirica, Craig Schofield, Samantha McLean, Katherine Landwehr, Nigel Farrow, Eugene Roscioli, David Parsons, Darryl A. Knight, Christopher Grainge, Andrew T. Reid, Su-Kim Loo, Punnam C. Veerati.
