# Supplementary material for: Dysregulated Notch Signaling in the Airway Epithelium of Children with Wheeze
Source: J Pers Med. 2021 Dec 7;11(12):1323. doi: 10.3390/jpm11121323 (PMC8707470; doi:10.3390/jpm11121323)
Supplement: Supplementary file 1 [file jpm-11-01323-s001.zip › SupplementaryMaterials_JPM_TIosifidis_CleanVersion_v3.pdf]

Supplementary Materials

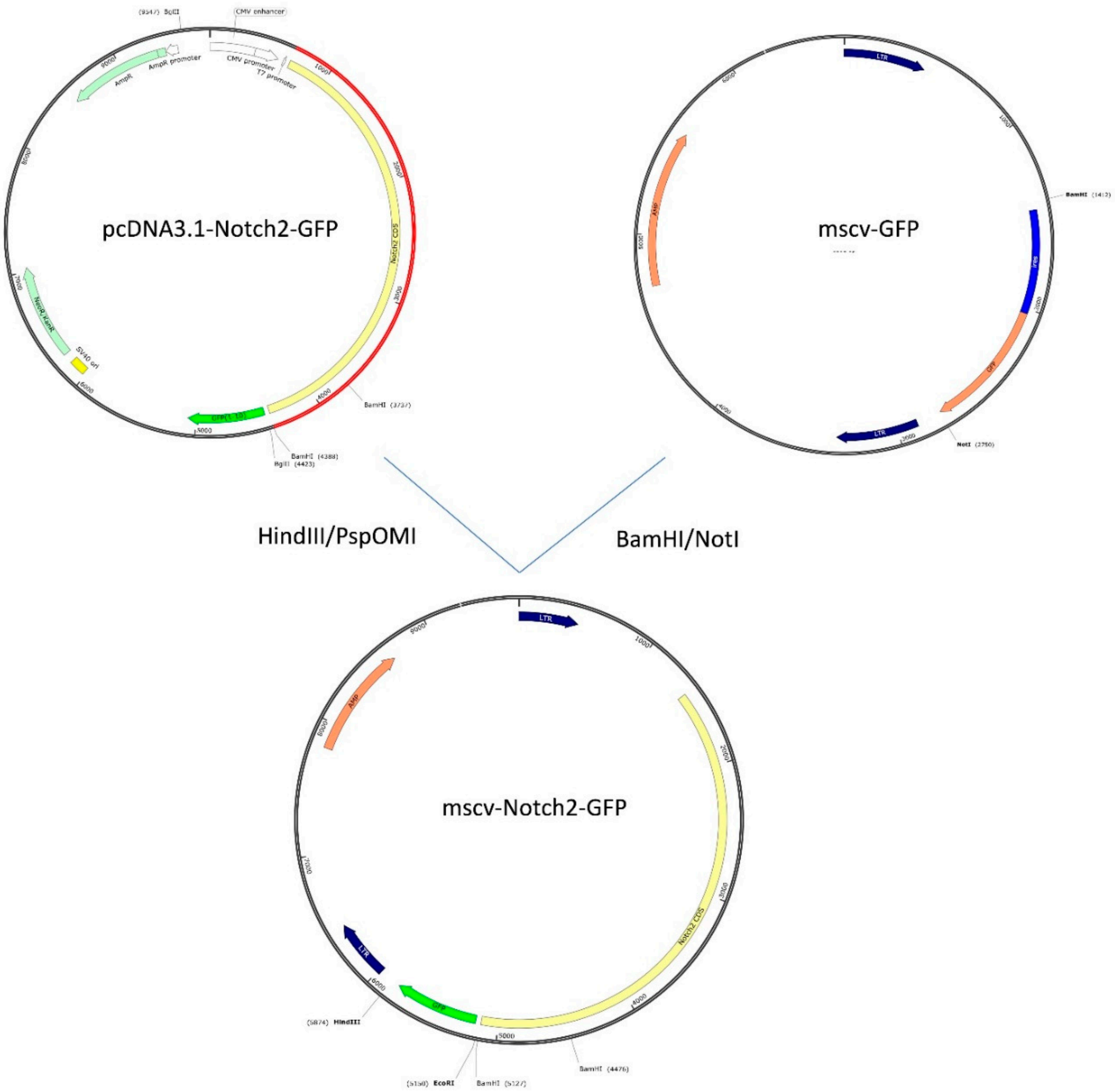

Figure S1. Preparation of MSCV plasmid for Notch2 overexpression studies.

The MSCV-IRES-GFP plasmid was used as a recipient plasmid following BamHI and NotI restriction digest to excise the IRES-GFP fragment. The Notch2.GFP gene was excised from the donor pcDNA3.1 Notch2.GFP plasmid by HindIII and PspOMI restriction digest. The Notch2 fragment was then gel purified, extracted using QIAquick kit and ligated to the MSCV plasmid at a ratio of 3:1 using T4 DNA ligase. Plasmid sequences were confirmed by DNA sequencing.

A

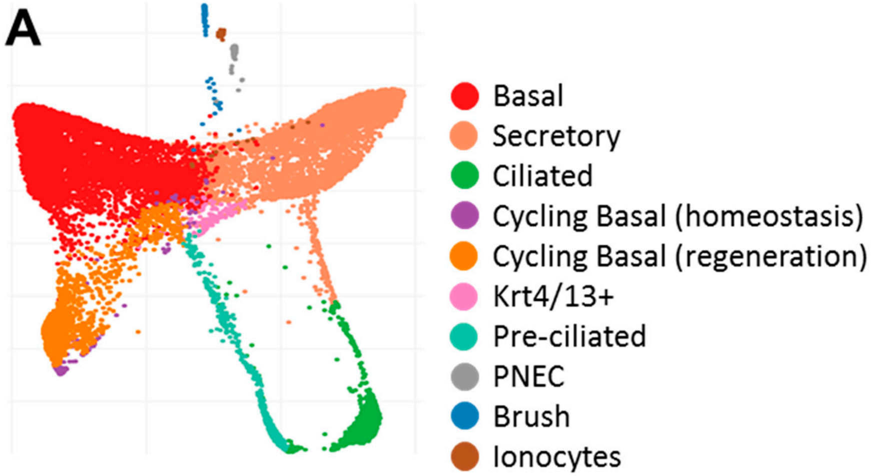

	Events	%Proportion
**Basal**	6009	42.4
**Secretory**	4792	33.8
**Ciliated**	1333	9.4
**Cycling Basal (h)**	1242	8.8
**Cycling Basal (r)**	128	0.9
**KRT4/13+**	117	0.8
**Pre-ciliated**	366	2.6
**PNEC**	74	0.5
**Brush**	73	0.5
**Ionocytes**	29	0.2
**TOTAL**	14163	100.0

B

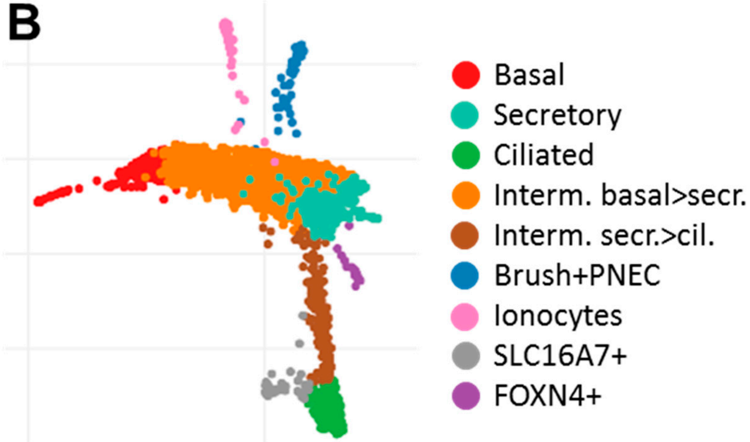

	Events	%Proportion
**Basal**	252	8.5
**Secretory**	280	9.4
**Ciliated**	258	8.7
**Inter. basal>secr**	1812	61.0
**Inter. secr. > cil.**	202	6.8
**Brush+PNEC**	56	1.9
**Ionocytes**	45	1.5
**SLC14A7+**	43	1.4
**FOXN4+**	22	0.7
**TOTAL**	2970	100.0

Figure S2. Cellular composition of airway epithelium.

(A-B) Publicly available single cell RNA-Sequencing data from mouse (A) and human (B) tracheobronchial epithelial cells were mined to identify cellular composition of airway epithelium (e.g., basal, secretory, ciliated, etc.). Number of airway epithelial cells and cell subset proportion (% total) are presented.

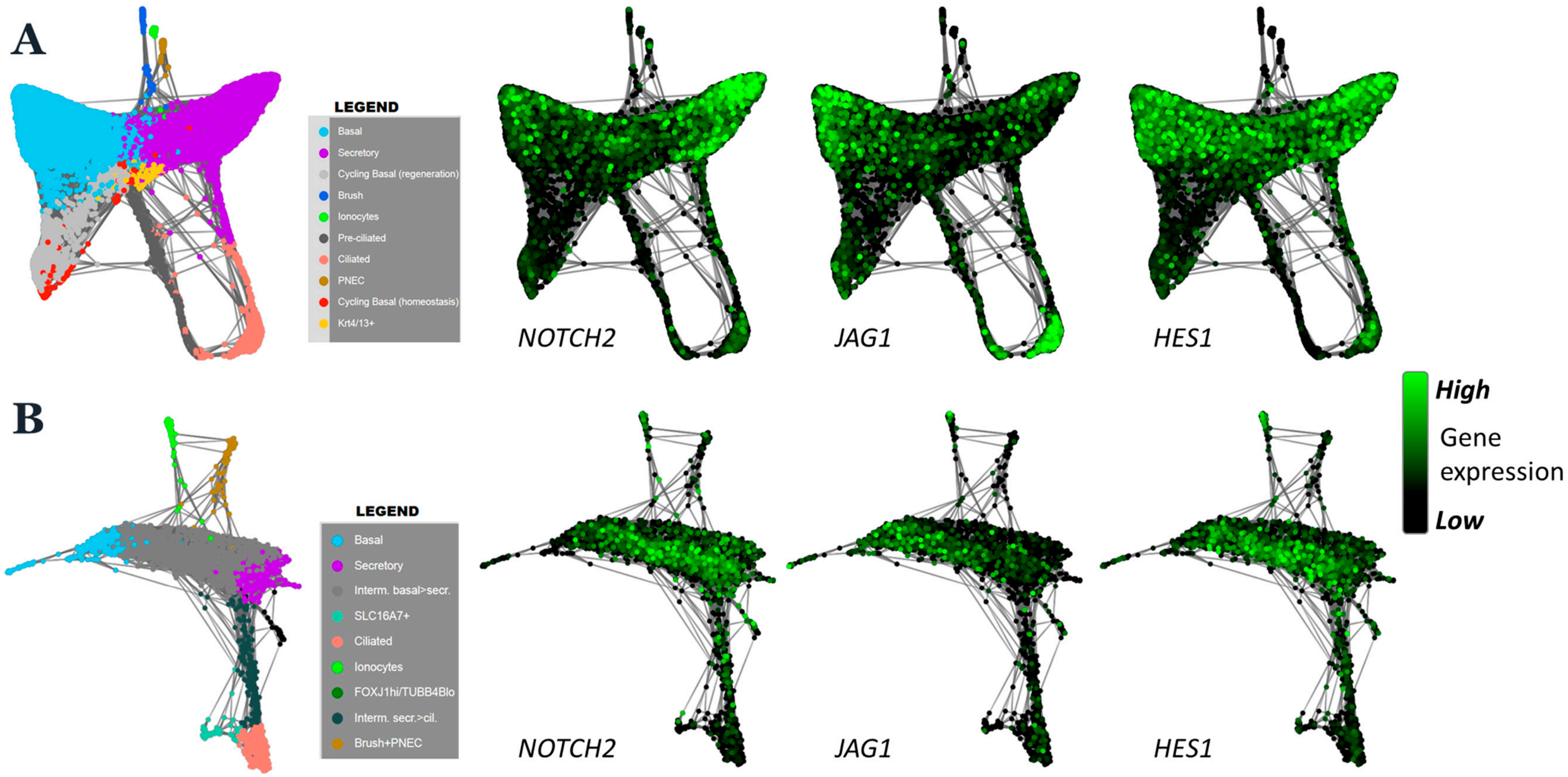
A
LEGEND
Basal
Secretory
Cycling Basal (regeneration)
Brush
Ionocytes
Pre-ciliated
Ciliated
PNEC
Cycling Basal (homeostasis)
Krt4/13+
NOTCH2
JAG1
HES1
B
LEGEND
Basal
Secretory
Interm. basal>secr.
SLC16A7+
Ciliated
Ionocytes
FOXJ1hi/TUBB4Blo
Interm. secr.>cil.
Brush+PNEC
NOTCH2
JAG1
HES1
High
Gene expression
Low

Figure S3. Notch gene expression profiles in airway epithelial cell subsets.

(A-B) Publicly available single cell RNA-Sequencing data from mouse (A) and human (B) tracheobronchial epithelial cells were mined to identify Notch gene expression in each airway epithelial cell subset (e.g., basal, secretory, ciliated, etc.). Specifically, gene expression of *NOTCH2*, *JAG1* and Notch signaling downstream target, *HES1*, were interrogated. Black color indicates no gene expression and green is high gene abundance.

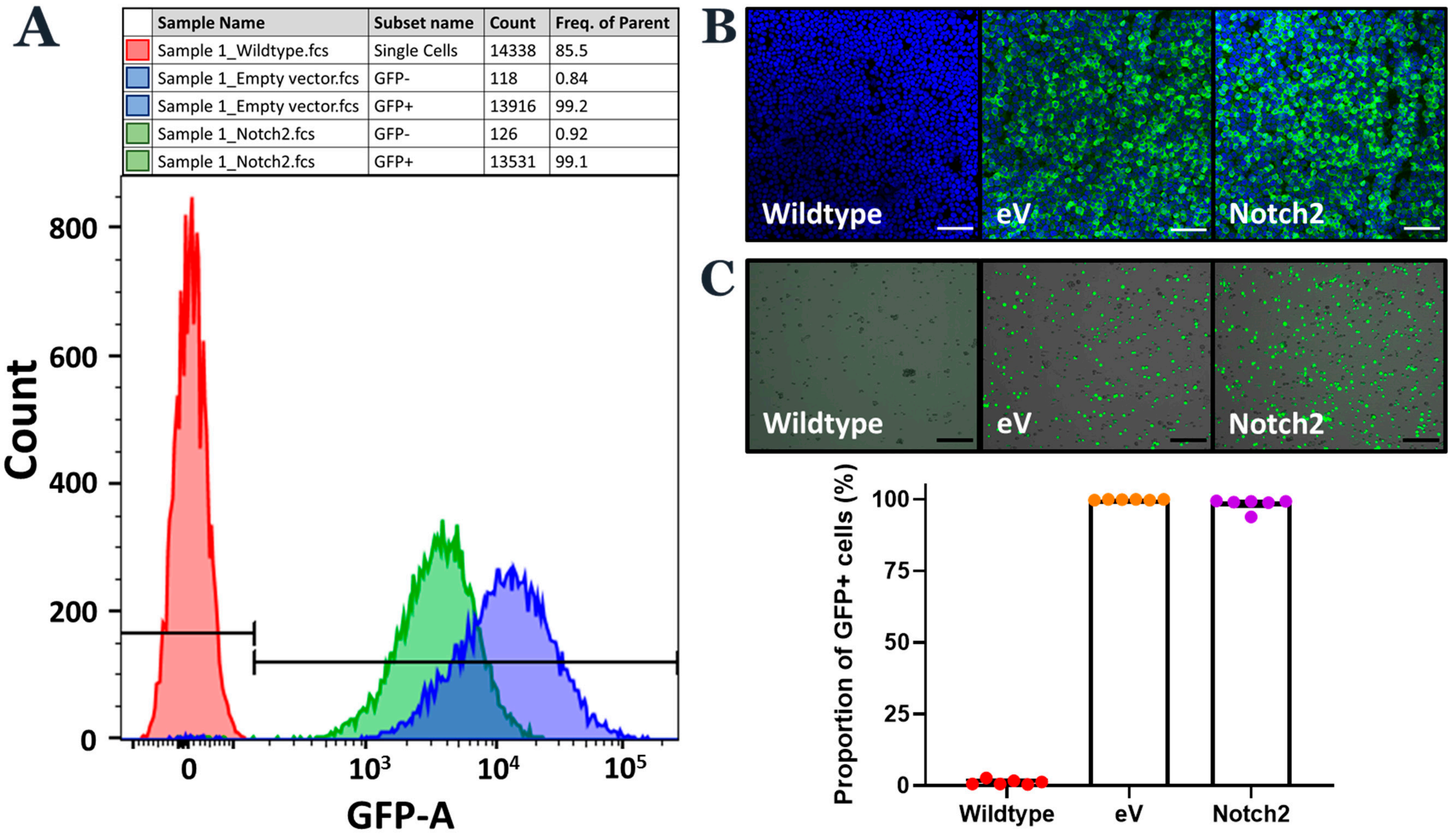
A
Sample Name
Subset name
Count
Freq. of Parent
Sample 1_Wildtype.fcs
Single Cells
14338
85.5
Sample 1_Empty vector.fcs
GFP-
118
0.84
Sample 1_Empty vector.fcs
GFP+
13916
99.2
Sample 1_Notch2.fcs
GFP-
126
0.92
Sample 1_Notch2.fcs
GFP+
13531
99.1
Count
800
600
400
200
0
0
10^3
10^4
10^5
GFP-A
B
Wildtype
eV
Notch2
C
Wildtype
eV
Notch2
Proportion of GFP+ cells (%)
100
75
50
25
0
Wildtype
eV
Notch2

Figure S4. Confirmation of retroviral infection and integration of MSCV-Notch2-GFP or empty vector MSCV-GFP in pAEC from children with wheeze.

(A) Representative flow cytometry data from pAEC from one child with wheeze (total n=3 children with wheeze) showing efficient overexpression of MSCV-Notch2-GFP (99.1% positive) or empty vector MSCV-GFP (99.2% positive) compared to uninfected wildtype control cultures. (B) Fluorescent imaging of pAEC cultures (empty vector and Notch2) identified most cells to express GFP (green) and no GFP signal in the wildtype control cells. Cell nuclei were counterstained with DAPI (blue). Scale bar: 100 µm (magnification 20x). (C) Cell counts were performed using the Tali™ Image-Based Cytometer with GFP-expressing cells shown in green. The empty vector (orange) and Notch2 (purple) overexpressing pAEC cultures included >95% GFP positive cells and wildtype (red) pAEC cultures were GFP negative as expected. Scale bar: 200 µm (magnification 10x). Data was generated from n=6 children with wheeze.

Study participants demographics

Table S4. Study participants demographics.

	Non-wheeze	Wheeze
**Number**	26	20
**Male (Female)**	13 (13)	17 (3)
**Mean age (range), years**	5.3 (1.2-15.6)	5.8 (2.6-13.5)

Table S5. Description of study participants demographics for all figures.

Study Participant Number	Respiratory Wheeze Status	Sex (M/F)	Age (years)	Figures(s)
1	Non-wheeze	F	7.1	2,3
2	Non-wheeze	F	5.2	2,3
3	Non-wheeze	M	7.9	2,3
4	Non-wheeze	F	10.1	2,3
5	Non-wheeze	F	5.6	2,3
6	Non-wheeze	F	1.9	2,3
7	Non-wheeze	M	1.7	2,3
8	Non-wheeze	M	9.6	2,3
9	Non-wheeze	M	6.9	4
10	Non-wheeze	F	10.1	4
11	Non-wheeze	F	5.6	4
12	Non-wheeze	F	5.8	4
13	Non-wheeze	F	5.8	4
14	Non-wheeze	F	9.4	4
15	Non-wheeze	M	15.6	5A
16	Non-wheeze	F	1.2	5A
17	Non-wheeze	M	2.9	5A

18	Non-wheeze	M	1.9	5A
19	Non-wheeze	M	2.3	5A,5B
20	Non-wheeze	M	2.1	5B
21	Non-wheeze	M	3.7	5B
22	Non-wheeze	M	3.0	5B,6
23	Non-wheeze	M	3.2	6
24	Non-wheeze	F	2.6	6
25	Non-wheeze	F	2.9	6
26	Non-wheeze	M	2.8	5B,6
27	Wheeze	F	4.2	2,3
28	Wheeze	M	6.9	2,3
29	Wheeze	M	4.0	2,3
30	Wheeze	M	5.4	2,3
31	Wheeze	M	6.9	2,3
32	Wheeze	M	11.0	2,3
33	Wheeze	M	13.5	2,3
34	Wheeze	M	3.8	2,3
35	Wheeze	M	6.9	4
36	Wheeze	F	8.9	4
37	Wheeze	M	3.3	4
38	Wheeze	M	4.6	4
39	Wheeze	M	9.8	4
40	Wheeze	M	4.9	4
41	Wheeze	M	5.2	5,6
42	Wheeze	M	3.2	5,6
43	Wheeze	M	3.1	5,6
44	Wheeze	M	4.0	5,6
45	Wheeze	M	2.9	5,6
46	Wheeze	F	2.6	5,6

Gene expression analysis

Table S6. Details of reference and target genes. List of all forward/reverse primer sets used in a Sybr Green-based qPCR. *Housekeeping reference gene.

Gene symbol	Gene name	Primer set	Amplicon length (bp)
*18S**	18S ribosomal RNA	Forward: TAACCCGTTGAACCCCATTC<br>Reverse: TCCAATCGGTAGTAGCGACG	110
NOTCH1	Notch receptor 1	Forward: GGTCAATGCGAGTGGC<br>Reverse: GGCAGCAAGGCTACTGTG	153
NOTCH2	Notch receptor 2	Forward: ACTTCCTGCCAAGCATTCC<br>Reverse: GTCCATGTCTTCAGTGAGAAC	346
NOTCH3	Notch receptor 3	Forward: TTCTTAGATCTTGGGGGCCT<br>Reverse: GGAAGAAGGAGGTCCCAGAC	238
NOTCH4	Notch receptor 4	Forward:<br>TTTATTATGGGTGACAGATTTAGGG<br>Reverse: CACCCCCGCTGATGACTC	126
JAG1	Jagged 1	Forward: AGCAGTATTCACACTTGCTGTTG<br>Reverse: TGAAAGGCTTTTCAACCACA	134
JAG2	Jagged 2	Forward: AGAAGACTGCAACAGCTGCC<br>Reverse: AACAGACCTGTGGAAGAGCC	591

DLL1	Delta-like 1	Forward: CAAGGATATATGCCCCAACG<br>Reverse: CAGGCAGTGCATGCTTCTTA	250
DLL3	Delta-like 3	Forward: GAGACACCCAGGTCCTTTGA<br>Reverse: CAGTGGCAGATGTAGGCAGA	152
DLL4	Delta-like 4	Forward: AAGGGCCTAGACCAGAGAGC<br>Reverse: CTGGGTGCGTCTCTTGCT	122
